# Extracorporeal Shockwave Therapy Modulates the Expressions of Proinflammatory Cytokines IL33 and IL17A, and Their Receptors ST2 and IL17RA, within the Articular Cartilage in Early Avascular Necrosis of the Femoral Head in a Rat Model

**DOI:** 10.1155/2021/9915877

**Published:** 2021-07-09

**Authors:** Jai-Hong Cheng, Shun-Wun Jhan, Chieh-Cheng Hsu, Hung-Wen Chiu, Shan-Ling Hsu

**Affiliations:** ^1^Center for Shockwave Medicine and Tissue Engineering, Kaohsiung Chang Gung Memorial Hospital and Chang Gung University College of Medicine, Kaohsiung 833, Taiwan; ^2^Medical Research, Kaohsiung Chang Gung Memorial Hospital and Chang Gung University College of Medicine, Kaohsiung 833, Taiwan; ^3^Department of Leisure and Sports Management, Cheng Shiu University, Kaohsiung 833, Taiwan; ^4^Department of Orthopedic Surgery, Sports Medicine, Kaohsiung Chang Gung Memorial Hospital and Chang Gung University College of Medicine, Kaohsiung 833, Taiwan; ^5^Fooyin University, School of Nursing, Kaohsiung 831, Taiwan

## Abstract

Avascular necrosis (AVN) of the femoral head (AVNFH) is a disease caused by injury to the blood supply of the femoral head, resulting in a collapse with osteonecrosis and damage to the articular cartilage. Extracorporeal shockwave therapy (ESWT) has been demonstrated to improve AVNFH owing to its anti-inflammation activity, angiogenesis effect, and tissue regeneration in clinical treatment. However, there are still so many pieces of the jigsaw that need to be fit into place in order to ascertain the mechanism of ESWT for the treatment of AVNFH. The study demonstrated that ESWT significantly protected the trabecular bone volume fraction BV/TV (*P* < 0.01) and the trabecular thickness (*P* < 0.001), while in contrast, the trabecular number and trabecular separation were not significantly different after treatment as compared with AVNFH. ESWT protected the articular cartilage in animal model of AVNFH. The levels of IL1-*β* and IL33 were significantly induced in the AVNFH group (*P* < 0.001) as compared with Sham and ESWT groups and reduced in ESWT group (*P* < 0.001) as compared with AVNFH group. In addition, the expression of the receptor of IL33, ST2, was reduced in AVNFH and induced after ESWT (*P* < 0.001). The expression of IL17A was induced in the AVNFH group (*P* < 0.001) and reduced in the ESWT group (*P* < 0.001). Further, the expression of the receptor of IL17A, IL17RA, was reduced in the AVNFH group (*P* < 0.001) and improved to a normal level in the ESWT group as compared with Sham group (*P* < 0.001). Taken together, the results of the study indicated that ESWT modulated the expression of IL1-*β*, pro-inflammatory cytokines IL33 and IL17A, and their receptors ST2 and IL17RA, to protect against loss of the extracellular matrix in the articular cartilage of early AVNFH.

## 1. Introduction

Avascular necrosis (AVN) or osteonecrosis of the femoral head (AVNFH) is a major, painful hip joint disorder that causes severe hip disability, requiring total hip arthroplasty (THA). The quality of life of patients is seriously affected by this disease, especially in younger patients [1]. Although the survival rate of THA patients has improved over the past few years, its durability is still limited, and treatment for joint preservation is preferred. Traditional treatment for AVNFH is recommended within the early stages and includes nonsteroid drugs, protected weight-bearing, and physical treatments, but the outcomes are often disappointing [2, 3]. Surgical interventions in symptomatic hips are preceded by core decompression, muscle pedicle grafts, nonvascularized or vascularized bone grafts, and derotational osteotomy [4–7]. However, to date, the outcomes of all methods are varying and unreliable; therefore, a new and effective treatment for AVNFH is needed.

Pathologically, AVNFH is characterized by the destruction of blood flow to the femoral head, which induces bone damage and necrosis [8, 9]. If the healing process of reparative tissue from necrosis of bone does not overcome the damage caused, further collapse of the head and joint in the femur could occur [10]. Prevention of progressive degradation of AVNFH is difficult. Core decompression is a well-known technique that has been used to treat AVNFH for more than three decades [11], and core decompression and avascular or vascularized bone grafting have been demonstrated to achieve good and moderate results for early AVNFH. However, the variability of core decompression in terms of the clinical success rate was reported to be only 63%, and the rate of subsequent joint replacement surgery or hip salvage surgery was reported to be approximately 33% of patients [12–14]. Complications of core decompression include donor site morbidity and nerve palsy [15]. One physical method, a noninvasive treatment, is extracorporeal shockwave therapy (ESWT), which has been demonstrated to be safe for the treatment of AVNFH.

ESWT has been shown to be effective in the treatment of musculoskeletal disorders, including nonunion and tendinopathy of the shoulder, elbow, knee, and heel [16–22]. Many studies have reported that ESWT also exerts beneficial effects in osteonecrosis. There are many varieties of growth factors and tissue repair factors that are induced by ESWT, such as vascular endothelial growth factor (VEGF), insulin-like growth factor- (IGF-) I, transforming growth factor- (TGF-) beta, epithelial growth factor (EGF), fibroblast growth factor (FGF), platelet-derived growth factor (PDGF), proliferating cell nuclear antigen (PCNA), von Willebrand factor (vWF), Wnts, endothelial nitric oxide synthase (eNOS), and osteocalcin and bone morphogenetic proteins (BMPs) [19, 20, 23, 24]. The results of animal studies have shown that ESWT promotes bone remodeling and tissue regeneration with ingrowth of angiogenic and osteogenic growth factors [24, 25]. Recently, many clinical studies have revealed that ESWT appears to be effective for the treatment of early AVNFH [20]. ESWT is reported to result in clinical improvement in 79% of AVNFH patients; however, only 39% of patients achieved regression of the lesion according to magnetic resonance imaging (MRI) [26]. The results showed that twenty-three patients with stage I, II, or III lesions treated with ESWT and seven patients for whom THA was performed due to failure of treatment saw no improvement, or worsened. Another long-term follow-up study of the outcomes of ESWT for early AVNFH revealed that the necessity for THA increased with time, and 24% (7 of 29) of patients underwent surgery at 8-9 years after ESWT [27]; three patients (four hips) received a second course of shockwave treatment, and three hips eventually underwent THA. Therefore, most clinic studies demonstrated that ESWT is more effective than surgical intervention in early phase treatment.

In recent studies, pro-inflammatory cytokines, interleukin 33 (IL33), and interleukin 17A (IL17A), which are members of the interleukin 1 (IL1) family, play roles in osteonecrosis [28, 29]. Expressions of IL33 and IL17A have been observed in the serum and inflamed synovium of AVNFH patients [29, 30]. These results indicate that IL33 and IL17A may be involved in the development of this disease and could represent treatment targets. ESWT has been reported to exert immunomodulatory effects in the inflammatory disease [23, 31, 32]. In this study, we attempted to elucidate the expressions of pro-inflammatory cytokines IL33 and IL17A, and their receptors ST2 and IL17RA, in the articular cartilage of the animal model of AVNFH after ESWT.

## 2. Materials and Methods

### 2.1. Animals

The twenty-four rats were obtained and treated humanely according to the Guide for the Care and Use of Laboratory Animals. The IACUC protocol of the animal study was approved by the Animal Care Committee of Kaohsiung Chang Gung Memorial Hospital, and the approval number was 2019031801. The animals were maintained and cared for before and after the experiments in the Center for Laboratory Animals; they were housed at 23 ± 1°C with a 12-hour light and dark cycle and given food and water.

### 2.2. Study Design

**Figure 1:**
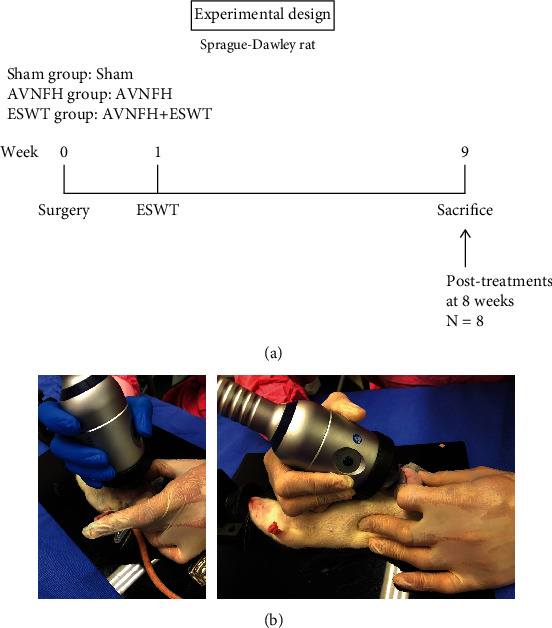
The study design and application of shockwave therapy. (a) The graph displayed the study design of the experiment, including ONFH surgery, shockwave application, and sacrificed animals. (b) The two focal points were approximately 0.5 cm apart and the corresponding locations on the skin in the groin area to make with a marker. Each of the two points was treated with 2000 impulses of shockwaves at 0.25 mJ/mm^2^ energy flux density and total of 4000 impulses of shockwaves were applied to the affected femoral head. *N* = 8 for all groups.

The twenty-four rats were randomized into three groups for experiments (Figure 1(a)). The Sham group was the sham control, without surgery or treatment. In the AVNFH group, AVNFH was induced in the rats by anterior hip arthrotomy, transection of the ligamentum teres and vascular deprivation of femoral neck by electrocoagulation on left hips. Finally, in the ESWT group, AVNFH rats received shockwave therapy (0.25 mJ/mm^2^ with 4000 impulses, 4 Hz) to the neck of left femur one week postsurgery. All rats were sacrificed at 9 weeks post-surgery.

### 2.3. Avascular Necrosis of Femoral Head Rat Model

Sprague-Dawley rats (night weeks of age, 220 g) were anesthetized using Zoletil (25 mg/kg) and Xylazine (10 mg/kg). The left hips of rats were subjected to surgery and opened the capsule without remove the muscles by comprising anterior hip arthrotomy. The ligamentum teres of hips were transected and periosteum was removed carefully. The blood vessel around the femoral neck was deprived by using electrocoagulation to induce AVNFH. The ampicillin (25 mg/kg) and ketorolac (1 mg/kg/day) were administered to prevent infection and reduce pain for 5 days after surgery. All rats were allowed unrestricted weight-bearing and activity.

### 2.4. Shockwave Treatment

The animals in the AVNFH group that received shockwave therapy at one week postsurgery comprised the ESWT group. Shockwaves were generated using a DUOLITH SD1 device (Storz Medical AG, Tägerwilen, Switzerland). We selected two focal points, approximately 0.5 cm apart, and the corresponding locations on the skin in the groin area were marked with a marker pen. Each of the two points were subjected to 2000 impulses of shockwaves at an energy flux density of 0.25 mJ/mm^2^, and in total, 4000 impulses of shockwaves were applied to the affected femoral head, as shown in Figure 1(b).

### 2.5. Micro-CT Analysis

Harvested lower-limb specimens were subjected to micro-CT scanning (SkyScan, 1176, Kartuizersweg 3B 2550 Kontich, Belgium): filter A1 0.5 mm, exposure 270 ms, isotopic pixel size 18 × 18 × 18 *μ*m, X-ray voltage 50 kV, 500 *μ*A. The left hip of the rat was prepared and sized prior to micro-CT for scanning. Image reconstruction was performed, and a series of planar transverse grey images were generated using NRecon software (Skyscan). The region of interest (ROI) of the bone morphometry was selected, and the trabecular volume fraction (BV/TV), trabecular thickness (Tb.Th), trabecular number (Tb.N), and trabecular separation (Tb.Sp) were obtained using the Skyscan CT-analyser program.

### 2.6. Histopathological Examination

Specimens were performed for histopathological examination. The left hips of the rats were fixed in 4% PBS-buffered formaldehyde at 4°C for one day and decalcified in 10% PBS-buffered EDTA at 4°C for one month. Decalcified hips were fixed and embedded to paraffin wax, and sliced into 5-*μ*m-thick sections. The samples were then stained with hematoxylin-eosin (HE) and safranin-O. The level of damage to the degenerative cartilage was assessed from the results of safranin-O staining using the Osteoarthritis Research Society International (OARSI) cartilage OA grading system; scores were obtained on a 0-to-24 scale by multiplying the index of the grades with the stage.

### 2.7. Immunohistochemical Analysis

The articular cartilage of femur heads was further analyzed with specific antibodies for immunohistochemical analysis, as follows: IL1-*β* (Abcam, USA, Ab-9787, 1 : 200), IL33 (Biorbyt, USA, orb6205, 1 : 200), ST2 (Proteintech, USA, 11920-1-AP, 1 : 150), type II collagen (Santa Cruz Biotechnology, USA, Sc-52658, 1 : 100), IL17A (Invitrogen, USA, PA5-79470, 1 : 200), and IL17RA (Abcam, USA, ab218249, 1 : 200). Sections of the samples were probed with specific proteins for anti-rat IL1-*β*, IL33, ST2, type II collagen, IL17A, and IL17RA to identify protein markers in the articular cartilage of the rats. The immunoreactivity of samples was assessed using a HRP-DAB Cell and Tissue Staining Kit (R & D Systems, USA). The immunoactivities were quantified from five areas in three sections of the same specimen using a Zeiss Axioskop II plus microscope (Carl Zeiss, Germany). Images were captured using a Cool CCD camera (SNAP-Pro c.f. Digital kit; Media Cybernetics, USA) and analysed using the Image-Pro® Plus software (Media Cybernetics, USA). The percentage of positive signals in each area was calculated, and the average of each sample was used as the result for analysis.

### 2.8. Statistical Analysis

Statistical software SPSS version 17.0 (SPSS Inc., Chicago, IL, USA; http://www.ibm.com/tw-zh/analytics/spss-trials) was employed for statistical analysis. Differences and significances of differences between groups were compared using one-way ANOVA for parametric data with *P* < 0.05, *P* < 0.01, and *P* < 0.001.

## 3. Results

### 3.1. ESWT Protected the Subchondral Bone in an Early AVNFH Rat Model

**Figure 2:**
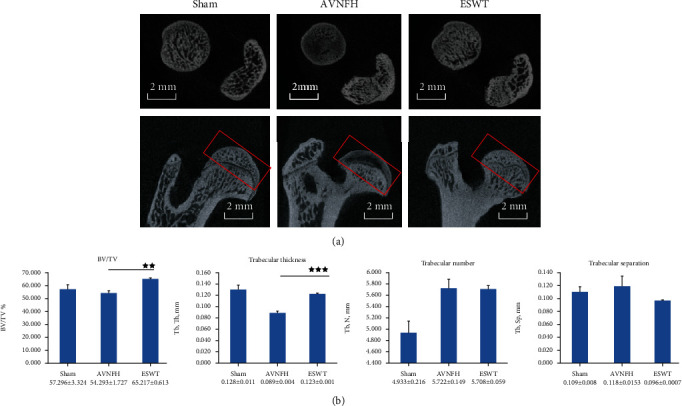
Micro-CT scan of the left femur of subchondral bone in different groups. (a) The results showed photomicrographs of the femur head in sagittal and transverse views from micro-CT. The region of interesting was indicated by a red rectangle. (b) The data of subchondral bone of femur head displayed the graphic illustrations of the trabecular bone volume fraction (BV/TV), trabecular thickness, trabecular number, and trabecular separation.^★★^*P* < 0.01 and ^★★★^*P* < 0.001 as compared with the AVNFH group. The scale bar was 2 mm. *N* = 8 for all groups.

In the experiments, we established a rat AVNFH model and applied ESWT to the femur head of AVNFH rat to establish the ESWT group (Figure 1). The rats in each group were sacrificed posttreatment at 8 weeks. Via micro-CT scanning, the subchondral bone of the left femur head was observed to have been protected against damage after ESWT as compared with the AVNFH group (Figure 2(a), sagittal and transverse views). The results showed that ESWT protected the damage of bone in the femur head of AVNFH.

Micro-CT data showed that ESWT significantly increased the trabecular bone volume fraction BV/TV (*P* < 0.01) and trabecular thickness (*P* < 0.001) in the subchondral bone of the left femur head as compared with the AVNFH group (Figure 2(b)). The trabecular number and trabecular separation were not significantly different after ESWT.

### 3.2. ESWT Protected the Articular Cartilage in Early AVNFH

**Figure 3:**
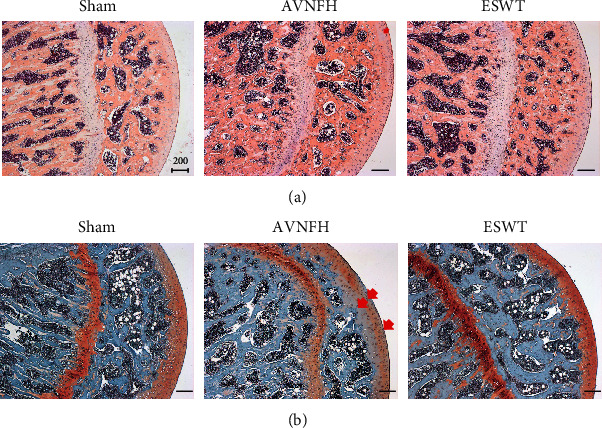
The microphotographs of the left femur head showed the changes of articular cartilage in the Sham, AVNFH, and ESWT groups. Some areas of disorganization, loss of extracellular matrix, and decreased number of chondrocytes are presented (arrow) and are protected in the ESWT group by (a) hematoxylin-eosin and (b) safranin-O stain. The scale bar was 200 *μ*m. *N* = 8 for all groups.

**Figure 4:**
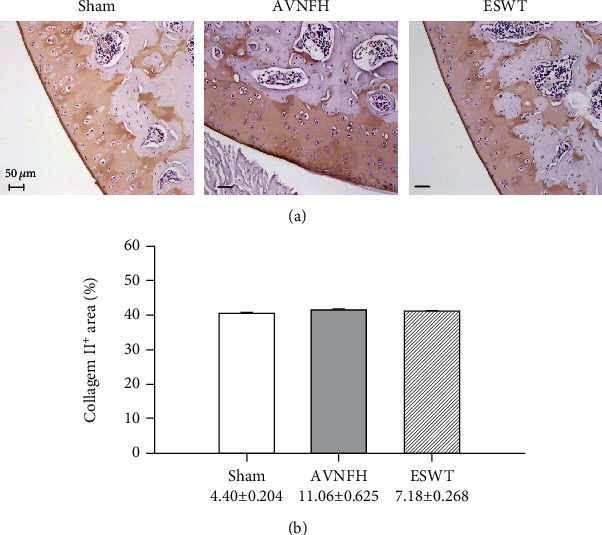
Immunohistochemical analysis for type II colagen in the articular cartilage of the left femur head (a) and the level of expression was measured after treatment (b). The scale bar was 50 *μ*m. *N* = 8 for all groups.

Pathological changes were measured using HE and safranin-O staining in the Sham, AVNFH, and ESWT groups (Figure 3). ESWT prevented the loss of the cellular matrix and chondrocytes of the articular cartilage of the hip joint as compared with the Sham and AVNFH groups posttreatment at 8 weeks according to the results of HE and safranin-O staining (Figures 3(a) and 3(b)). Some loss of cellular matrix tissues were observed in the articular cartilage of the AVNFH group as compared with Sham group (Figure 3(a), AVNFH group: red arrow). The recovered in the ESWT groups was obviously in safranin-O staining as compared with AVNFH group (Figure 3(b), AVNFH group: red arrow); however, the damage to cellular matrix tissue was not severe enough to increase the OARSI score greatly, and no significant difference was observed between the AVNFH and ESWT groups (Table 1). The expressed level of type II collagen was also measured in the Sham, AVNFH, and ESWT groups, and no significant differences were observed among the three groups (Figure 4). The results showed minor damages and pathological changes in the articular cartilage of AVNFH group at the end of the experiment duration, which recovered after ESWT.

### 3.3. ESWT Modulated the Expression of IL1-*β*, Th2-Oriented Cytokine IL33, and Receptor ST2 in the Articular Cartilage of Early AVNFH

**Figure 5:**
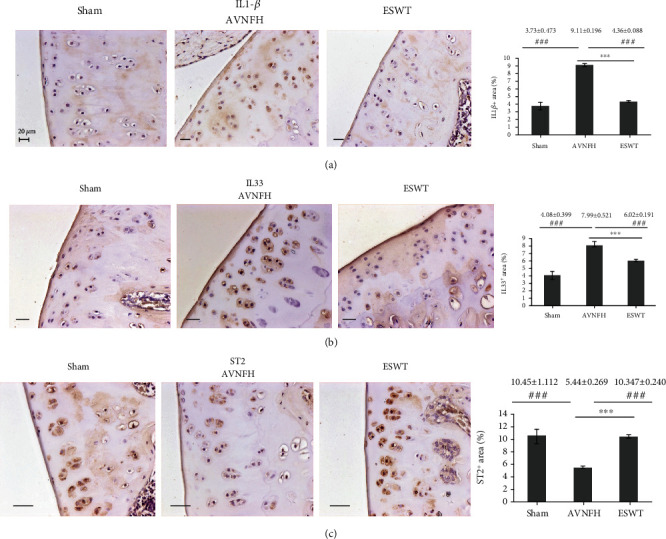
Immunohistochemical analysis for (a) IL1-*β*, (b) IL33, and (c) ST2 in the articular cartilage of the left femur head (right) and the level of expression was measured after treatment (left). ^∗∗∗^*P* < 0.001 as compared with ESWT group and ^###^*P* < 0.001 as compared with AVNFH group. The scale bar was 20 *μ*m. *N* = 8 for all groups.

In the experiment, the protein expression levels of IL1-*β*, IL33, and ST2 were surveyed by immunohistochemical analysis in the articular cartilage in the Sham, AVNFH, and ESWT groups (Figure 5). IL1-*β* and IL33 were significantly induced in the AVNFH group as compared with the Sham group and ESWT group (*P* < 0.001) and were reduced in the ESWT group as compared with the AVNFH group (*P* < 0.001). In addition, expression of the receptor of IL33, ST2, was reduced in the AVNFH group as compared with Sham and ESWT groups (*P* < 0.001) and increased after ESWT as compared with AVNFH (*P* < 0.001). The results demonstrated that ESWT modulates the inflammatory key factors IL1-*β*, Th2-oriented cytokine IL33, and receptor ST2 during cartilage repair in the treatment of AVNFH.

### 3.4. ESWT Modulated the Expression of pro-Inflammatory Cytokine IL17A and Receptor IL17RA in the Articular Cartilage of Early AVNFH

**Figure 6:**
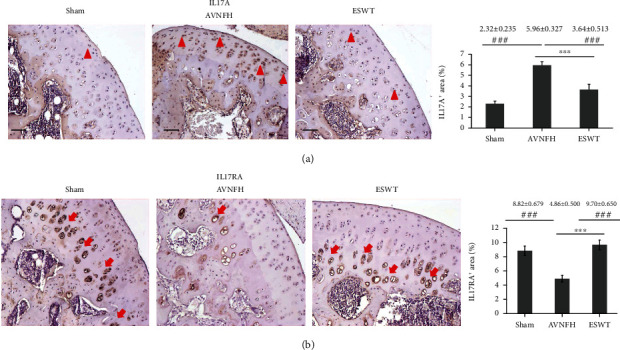
Immunohistochemical analysis for (a) IL17, (b) IL17RA in the articular cartilage of the left femur head (right), and the level of expression was measured after treatment (left). The expression of IL17A was major in superfacial zone and proliferation zone (arrowhead), while IL17RA was major expressed in the hypertrophic chondrocytes of the calcified cartilage zone (arrow). ^∗∗∗^*P* < 0.001 as compared with the ESWT group and ^###^*P* < 0.001 as compared with the AVNFH group. The scale bar was 50 *μ*m. *N* = 8 for all groups.

Immunohistochemical images displayed the levels of IL17A and receptor IL17RA in the articular cartilage of the Sham, AVNFH, and ESWT groups (Figure 6). The expression of IL17A was induced in the AVNFH group as compared with the Sham and ESWT groups (*P* < 0.001) and was reduced in the ESWT group as compared with AVNFH groups (*P* < 0.001). In addition, the receptor of IL17A, IL17RA, was obviously reduced in the AVNFH group as compared with the Sham and ESWT groups (*P* < 0.001) and improved to a normal level in the ESWT group as compared with AVNFH group (*P* < 0.001). These results demonstrated that ESWT modulates the key factors of pro-inflammation IL17A and receptor IL17RA for AVNFH cartilage repair.

## 4. Discussion

In the current study, ESWT for AVNFH induced modulation of pro-inflammatory cytokines and protection of the articular cartilage of the hip with avascularity of the femoral head. ESWT significantly protected the articular cartilage and subchondral bone in AVNFH. The results of this study displayed that the expressions of inflammatory cytokines IL1-*β*, IL33, and IL17A were induced in AVNFH and were reduced after ESWT; in contrast, the receptors of IL33 and IL17A, ST2 and IL17RA, were reduced in the AVNFH and were induced after ESWT. The expressions of IL1-*β*, IL33, and IL17A cytokines, and their receptors ST2 and IL17RA, were elucidated in the articular cartilage of the AVNFH rat model following ESWT.

In the clinical studies, ESWT was demonstrated to promote bone repair and protect the femoral head in early-stage osteonecrosis [20, 33]. ESWT has been shown to increase osteogenic factors such as bone morphogenic proteins (BMPs), osteocalcin, alkaline phosphatase, and insulin-like growth factor, as well as osteogenic transcription factors such as core-binding factor crl-l, Runt-related transcription factor 2, hypoxia-inducible factor 1-alpha, and vascular endothelial growth factor for bone remodeling [23, 24]. ESWT has also been shown to exert chondroprotective effects safely in animal models and in the clinical treatment of arthritis [25, 34]. The expressions of extracellular matrix proteins of the articular cartilage, including type II collagen, aggrecan, tenascin-C, and chitinase 3-like protein 1, are increased and the expression of the matrix metalloproteinases reduced after ESWT [25, 35]. In this study, the cellular matrix of the articular cartilage in AVNFH was protected by ESWT in a rat model (Figure 3). However, the collapse of the articular cartilage and repair in AVNFH rats after ESWT was not observed within the duration of this experiment; further study is required to elucidate and molecular mechanism of ESWT on the articular cartilage in AVNFH.

It has been reported that arthritis-related genes of IL1*β*, IL6, and TNF*α* are expressed in the cartilage in AVNFH and could be potential biomarkers for AVNFH [36]. IL33 is a member of the IL1 family, and the level of IL33 in the serum has been reported to be related to the progression of AVNFH [37]. IL33 has also been proposed to be a key molecule in arthritis [38]; however, the expression of IL33 and its receptor ST2 in the articular cartilage of AVNFH are still unclear. The expressions of IL33 and ST2 in the articular cartilage of AVNFH and after ESWT were elucidated in this study. In addition, a high level of IL33 induces the expression of inflammatory cytokines IL1-*β*, IL6, IL13, and IL17, as well as matrix metalloprotease- (MMP-) 3 and MMP-9 in arthritis [39]. The expression of ST2 affects the hypertrophic differentiation of chondrocyte and the expressions of hypertrophic markers such as Col X, OSC, VEGF, and MMP-13 [40]. Our results indicated that IL33 may induce an imbalance of cartilage anabolism in AVNFH, which is restored by ESWT. Further, ESWT could modulate the expression of ST2 to affect the function of hypertrophic chondrocyte in AVNFH. However, a detailed overview of the functions of IL33 and ST2 in the pathogenesis of AVNFH is still required for further investigation.

Recently, IL33 has been reported to be linked with IL17 in terms of contributing to immunological dysfunction in inflammatory diseases [41, 42]. The IL17 receptor is a complex that consists of IL17RA, IL17RB, IL17RC, IL17RD, and IL17RE [43]. IL17RA is a key component required for IL17A activity, and blocking of IL17 binding by IL17RA could inhibit the expression of IL-6 to prevent synovial inflammation in arthritis [44]. A high expression of IL17A contributes to cartilage degradation by inducing disintegrin-like and metalloproteinase with thrombospondin motifs (ADAMTS) protease and matrix metalloprotease in the articular cartilage in arthritis [43]. A high expression of IL17A was observed in the hyaline cartilage, and receptor IL17RA was evaluated with regards to hypertrophy chondrocyte (Figure 6). It has been reported that IL17A is expressed in a paracrine manner in proliferating chondrocytes and spreads out to prehypertrophic cells during fracture healing [45]. However, IL17 receptor is mainly expressed in prehypertrophic chondrocytes during bone fracture healing. The different localizations of expressions of IL17A and IL17RA may be due to the repair mechanisms in different diseases, such as bone fracture healing or cartilage regeneration. There are few studies of the functions of IL17 and IL17RA axial signaling in the articular cartilage in AVNFH and after ESWT, and additional studies are needed to further validate their functions. Finally, this study was the first to show that ESWT modulated the expressions of IL33 and IL17A and their receptors ST2 and IL17RA, for repairing articular cartilage defects in AVNFH.

## 5. Conclusions

ESWT has been reported to have a good efficacy and safety for the clinical treatment of early AVNFH [46]; however, the mechanism of ESWT in the treatment of AVNFH is still unclear, especially with regard to immunomodulation. The results of this study displayed that ESWT affected the repair of the subchondral bone and articular cartilage in an animal model of AVNFH. In addition, ESWT modulated the expressions of IL1-*β*, pro-inflammatory cytokines IL33 and receptor ST2, and IL17A and receptor IL17RA to protect against loss of the extracellular matrix in the articular cartilage of AVNFH.

## Figures and Tables

**Table 1:** OARSI score of the articular cartilage in Sham, AVNFH and ESWT groups.

	Average	Standard error	*P* value^∗^
Sham	0	0	0
AVNFH	1.28	0.19	*P* < 0.001
ESWT	1.08	0.13	*P* < 0.001

^∗^The *P* < 0.001 was as compared with the Sham group.

## Data Availability

The data used to support the findings of this study are included within the article.
